# Tn4661-mediated transfer of blaCTX-M-15 from Klebsiella michiganensis to an outbreak clone of Pseudomonas aeruginosa

**DOI:** 10.1099/mgen.0.001303

**Published:** 2024-10-16

**Authors:** Katelyn V. Bartlett, Ting L. Luo, Ana C. Ong, Rosslyn A. Maybank, William Stribling, Bernadette Thompson, Aubrey Powell, Yoon I. Kwak, Jason W. Bennett, Francois Lebreton, Patrick T. Mc Gann

**Affiliations:** 1 Multidrug-Resistant Organism Repository and Surveillance Network (MRSN), Walter Reed Army Institute of Research, Silver Spring, Maryland, USA; 2 Infection Prevention & Control, Brooke Army Medical Center, Joint Base San Antonio-Fort Sam Houston, Texas, USA; 3 Department of Pathology, Brooke Army Medical Center, Joint Base San Antonio-Fort Sam Houston, Texas, USA

**Keywords:** CTX-M, ESBL, nosocomial infection, *Pseudomonas aeruginosa*, Tn*4661*

## Abstract

Carriage of CTX-M-type extended-spectrum β-lactamase (ESBL) is rare in *Pseudomonas aeruginosa*. During routine surveillance of an endemic ST-621 *P. aeruginosa* at a large hospital, isolate MRSN 100690 carrying *bla*_CTX-M-15_ was cultured from a patient (P2). This was the first detection of this ESBL in the endemic ST-621 lineage. All 1 488 bacterial isolates collected from the same facility in the 12 months prior to the incidence of 100 690 were screened for the presence of *bla*_CTX-M-15_. A set of 183 isolates was identified, in which corresponding patient metadata was evaluated for spatiotemporal overlaps with P2. The resulting three isolates, along with 100 690, were long-read sequenced using the Oxford Nanopore MinION platform to determine a potential donor of *bla*_CTX-M-15_. The screen revealed a single *Klebsiella michiganensis* isolate, MRSN 895358, which carried an IncA/C2 plasmid harbouring *bla*_CTX-M-15_. Notably, the patient harbouring 895358, P1, occupied the same hospital room as P2 9 months prior. Genomic alignment revealed that both isolates shared an identical 80.8 kb region containing the IncA/C2 plasmid replicon and *bla*_CTX-M-15_. This region was plasmid bound in 895 358, but chromosomally bound in 100 690 due to Tn*4661*-mediated transposition. ESBL *bla*_CTX-M-15_ was acquired and subsequently integrated into the chromosome of a ST-621 *P. aeruginosa*, likely initiated by plasmid transfer from a *K. michiganensis* strain.

**Impact Statement**

*Pseudomonas aeruginosa* is a significant nosocomial threat worldwide due to its ability to persist in the hospital environment and to resist antimicrobial treatments. The latter is largely due to intrinsic resistance mechanisms, a hallmark of this species. Nevertheless, here we characterized a rare instance of an acquired extended-spectrum beta-lactamase (ESBL), usually found in Enterobacteriaceae, in an outbreak clone of *P. aeruginosa*. Only by leveraging both genomic and epidemiological data using electronic healthcare records could we reveal a possible origin, from a clinical isolate of *Klebsiella michiganensis*, and route for the interspecies transfer of this resistance gene. While originally plasmid-bound, analysis of complete genomic sequences revealed that the ESBL gene was chromosomal in *P. aeruginosa*, as a result of a transposition event. Because the donor and recipient isolates were collected from different hosts, the horizontal gene transfer likely happened within an environmental reservoir in the hospital. This was further supported by the fact that the two patients were hospitalized in the same intensive care unit room, in which the *P. aeruginosa* outbreak clone was recovered from a contaminated sink. This study underscores the value of genomic surveillance in detecting ‘strains’, ‘plasmids’ and ‘genes’ outbreaks in support of infection control strategies.

## Data Summary

Sequencing data have been deposited in the National Center for Biotechnology (NCBI) under BioProject PRJNA1129159.

## Introduction

The dissemination of extended-spectrum β-lactamases (ESBLs), which confer resistance to most β-lactam antibiotics, is a major public health concern limiting treatment options in healthcare settings [1]. In particular, the CTX-M family of ESBLs is widely disseminated, and has been found across multiple bacterial species, especially Enterobacterales [1]. This wide taxonomic reach is largely attributed to intra and interspecies horizontal plasmid transfer with assistance from insertion sequences (IS) and transposons (Tn) [2,3].

The presence of CTX-M is not restricted to Enterobacterales, with detection also being reported in non-fermentative Gram-negative bacilli such as *Acinetobacter baumannii* and *Pseudomonas aeruginosa* [4,5]. Multidrug-resistant (MDR) *Pseudomonas aeruginosa* is one of the most common healthcare-acquired infections (HAIs), and even in the absence of acquired antimicrobial resistance genes (ARGs) such as ESBLs, can be difficult to treat due to many intrinsic resistance mechanisms [6,7]. The increasing prevalence of ESBLs among *P. aeruginosa* populations represents a major concern as common anti-pseudomonal antibiotics, such as ceftazidime and cefepime, are rendered ineffective by these enzymes [8]. How these non-Enterobacterales acquire and maintain additional ARGs, especially the more potent loci such as ESBLs and carbapenemases, is critical for gaining a deeper understanding of the ARG dissemination.

In this study, we report a ST-621 *P. aeruginosa* (MRSN 100690) carrying a *bla*_CTX-M-15_ ESBL, cultured from a patient (P2) hospitalized in 2022. While it uniquely harboured *bla*_CTX-M-15_, 100 690 belongs to an outbreak clone, which spread for over two decades in this hospital, in part due to contaminated sink drains in patient’s rooms in the intensive care units [9]. An analysis of other *bla*_CTX-M-15_ carrying clinical and environmental isolates from the same facility revealed a ST-41 *Klebsiella michiganensis* (MRSN 895358), cultured from the blood of a separate inpatient (P1), that carried *bla*_CTX-M-15_ on a 207 .k kb IncA/C2 plasmid, 80.8 kb of which was found integrated into the *P. aeruginosa* chromosome. Crucially, both patients P1 and P2 were spatiotemporally linked, having shared the same hospital room 9 months apart.

## Methods

### Bacterial sample selection

The Multidrug Resistant Organism Repository and Surveillance Network (MRSN) collects and genetically characterizes MDR organisms across the US Military Health System [10]. As part of on-going surveillance, an endemic cluster of 258 ST-621 *Pseudomonas aeruginosa*, spanning over a decade, was identified at a single hospital (Facility A) [9]. Following identification of the cluster in 2019, extensive environmental sampling was conducted at Facility A and this strain was recovered from multiple sinks and drains across the entire building [9]. No CTX-M gene was detected in any ST-621 *P. aeruginosa* until January 2022 when MRSN 100690 was cultured from patient P2. To investigate the origins of the ESBL gene, a 12 month retrospective genomic screen of 1 488 isolates collected from Facility A in 2022 was conducted to identify isolates with *bla*_CTX-M-15_. A total of 183 candidate isolates were identified and the corresponding patient metadata was screened to determine spatial or temporal links to P2. The epidemiological screen yielded one isolate, *Klebsiella michiganensis* MRSN 895358, as the strongest candidate for the origins of *bla*_CTX-M-15_ in 100 690.

### Antibiotic susceptibility testing

Next. 100690 and 895 358 underwent antimicrobial susceptibility testing (AST) using a Vitek 2 (card GN AST 71 and GN ID; bioMérieux, NC, USA) in a College of American Pathologists (CAP)-accredited laboratory. MIC interpretations were guided using breakpoints set by the Clinical and Laboratory Standards Institute (CLSI) guidelines (34th Edition) [11]. Samples determined as intermediate (I) or resistant (R) were designated non-susceptible, and those determined sensitive (S) were designated susceptible.

### Quantitative real-time PCR (qPCR) analysis of the *bla*_CTX-M-15_ gene expression

RNA extraction and subsequent qPCR was conducted in triplicate on *K. michiganensis* MRSN 895358 and *P. aeruginosa* MRSN 100690 in the presence and absence of cefepime at a final concentration of 16 µg ml^−1^ as previously described [12]. Briefly, cells were grown in parallel to early-log phase (OD_600_=0.5) in Tryptic Soy Broth (TSB). One set was treated with cefepime (16 µg ml^−1^) and the other with sterile PBS for the final 15 min. RNA was stabilized with RNAprotect (Qiagen, Germantown, MD) and RNA extracted using the mirVANA miRNA Isolation Kit (Invitrogen). First-strand cDNA was synthesized on 250 ng of DNase-treated RNA using the iScript cDNA Synthesis Kit (Bio-Rad Laboratories, Hercules, CA), diluted to a final concentration of 5 ng µl^−1^, and 2 µl used for qPCR on a BioRad CFX96 real-time system (Bio-Rad Laboratories). All reactions were in 20 µl using SsoAdvanced Universal SYBR Green Supermix (Bio-Rad Laboratories). Each run contained appropriate no-reverse-transcriptase, negative, and no template DNA as controls. Transcripts from the housekeeping genes *rpoD* and *dnaB* were used to normalize gene expression (primer sequences can be found in the Supplementary Material, available with the online version of this article). One-way ANOVA with Tukey’s multiple-comparison correction was used to determine if *bla*_CTX-M-15_ transcript levels differed between the two strains.

### Whole-genome sequencing

Then, 100690 and 895 358 genomic DNA was extracted using the DNeasy UltraClean Microbial Kit (Hilden, Germany). Library construction was performed with the KAPA Library Quantification Kit (Roche Molecular Systems, Indianapolis, IN) for sequencing on an Illumina MiSeq benchtop sequencer (Illumina, San Diego, CA) as previously described [13]. Assemblies were generated *de novo* using Newbler v2.9 with minimum thresholds for contig size and coverage set at 200 bp and 49.5 x, respectively. Long reads were generated for both 100 690 and 895358 using a MinION (Oxford Nanopore Technologies, Oxford, UK) using the MinION Library Prep kit. Long-read data was assembled *de novo* with three assemblers (Flye v2.9.1, Miniasm v0.3, and Raven v1.8.1) and a consensus assembly was generated with Trycycler v0.5.3 [14]. Consensus assemblies were then polished with long reads using Medaka v1.7.1 (https://github.com/nanoporetech/medaka) and with short reads using Polypolish v0.5 [15].

### Bioinformatic analysis

*In silico* taxonomic identification, multi-locus typing (MLST), and ARG detection were performed as previously described [16]. Plasmid replicon genes were called using PlasmidFinder [17] and genomes were annotated with Bakta v1.8.2 [18]. Closed plasmid and chromosomal sequences from 100 690 and 895 358 were aligned with Mauve v2.4.0 [19] and visualized in Geneious Prime v2023.0.4 (https://www.geneious.com). Aligned sequences shared between both isolates were extracted and used as a reference for blast with publicly available closed genomes on NCBI. All available complete *P. aeruginosa* (*n*=667)*, K. pneumoniae* (*n*=2,053)*, K. oxytoca* (*n*=57)*, K. michiganensis* (*n*=51)*,* and *K. grimontii* (*n*=20) genomes on NCBI were downloaded in May 2023, and aligned to the reference using blast. Sequences with highest homology (alignment fraction and nucleotide identity) were selected for representation with EasyFig [20].

## Results

### Patient history revealed spatiotemporal link between P1 and P2

*Pseudomonas aeruginosa* MRSN 100690 was cultured from the sputum of an inpatient (P2) in Facility A 9 days after admission for acute respiratory distress syndrome (ARDS) due to COVID-19 (Table 1). The patient was undergoing extracorporeal membrane oxygenation (ECMO) at the time of culture and a nosocomial origin was strongly suspected. This isolate belonged to a large ST-621 cluster that was responsible for an unrecognized protracted outbreak spanning over a decade in this hospital with multiple links to contaminated drains and sinks, including the sink in this patient’s room [9]. However, unlike all previous ST-621 isolates collected, it carried the ESBL gene *bla*_CTX-M-15_.

**Table 1.** Basic characteristics of strains in this study

MRSN ID	Patient	Species*	MLST*	Culture type
895 358	P1	*K. michiganensis*	41	Blood
100 690	P2	*P. aeruginosa*	621	Sputum

1*In- silico derived Mmultilocus Ssequence Ttype (MLST).

**Fig. 1.**
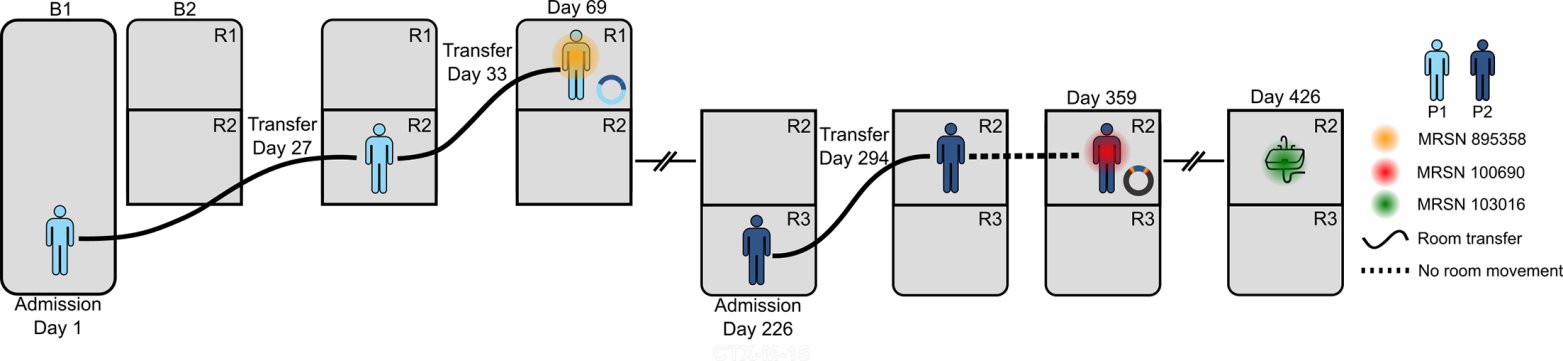
Spatiotemporal distribution of 100 690 and 895 358. A schematic following the movement of P1 (light blue) and P2 (dark blue) across the hospital ward following their initial intake. Hospital buildings are indicated as B1 or B2 and hospital rooms are indicated by black squares, labelled as R1, R2, or R3. Movement across rooms is indicated by a solid black line and continued admission in the same room by a dashed line. Culturing of 895358, 100 690, and 103 016 is indicated by orange, red, or green shading, respectively.

A screen of all 1 488 bacterial isolates from the same facility collected 12 months prior to the incidence of 100 690 were analysed for the presence of *bla*_CTX-M-15_. A result of 183 isolates carrying *bla*_CTX-M-15_ were identified, though only three had any spatiotemporal overlap with P2. These three isolates were identified as ST-41 *Klebsiella michiganensis*, ST-96 *Citrobacter portucalensis*, and Novel *Citrobacter freundii* complex. ST-41 *Klebsiella michiganensis* (MRSN 895358) was cultured from the blood of a different inpatient (P1) 9 months earlier (Table 1). This patient had been admitted for a prolonged stay 8 weeks earlier, strongly suggesting a nosocomial origin for the bloodstream infection. Further epidemiological investigations indicated that P1 and P2 had occupied the same room during their respective hospital stays (Fig. 1). Notably, genomic analysis revealed that both 100 690 and 895 358 carried an IncA/C2 replicon in association with *bla*_CTX-M-15_, suggesting that the *bla*_CTX-M-15_ in both isolates may have originated from the acquisition of a circulating plasmid. Other bacterial cultures from these patients were negative for *bla*_CTX-M-15_, indicating that only after occupancy of this shared room did P1 and P2 acquire isolates carrying *bla*_CTX-M-15_ embedded in an IncA/C2 background. Furthermore, ST-96 *C. portucalensis* and novel-ST *C. freundii* complex did not carry an IncA/C2 replicon, making them unlikely donors.

### Genomic structure surrounding *bla*_CTX-M-15_ in 100690 and 895358

**Fig. 2.**
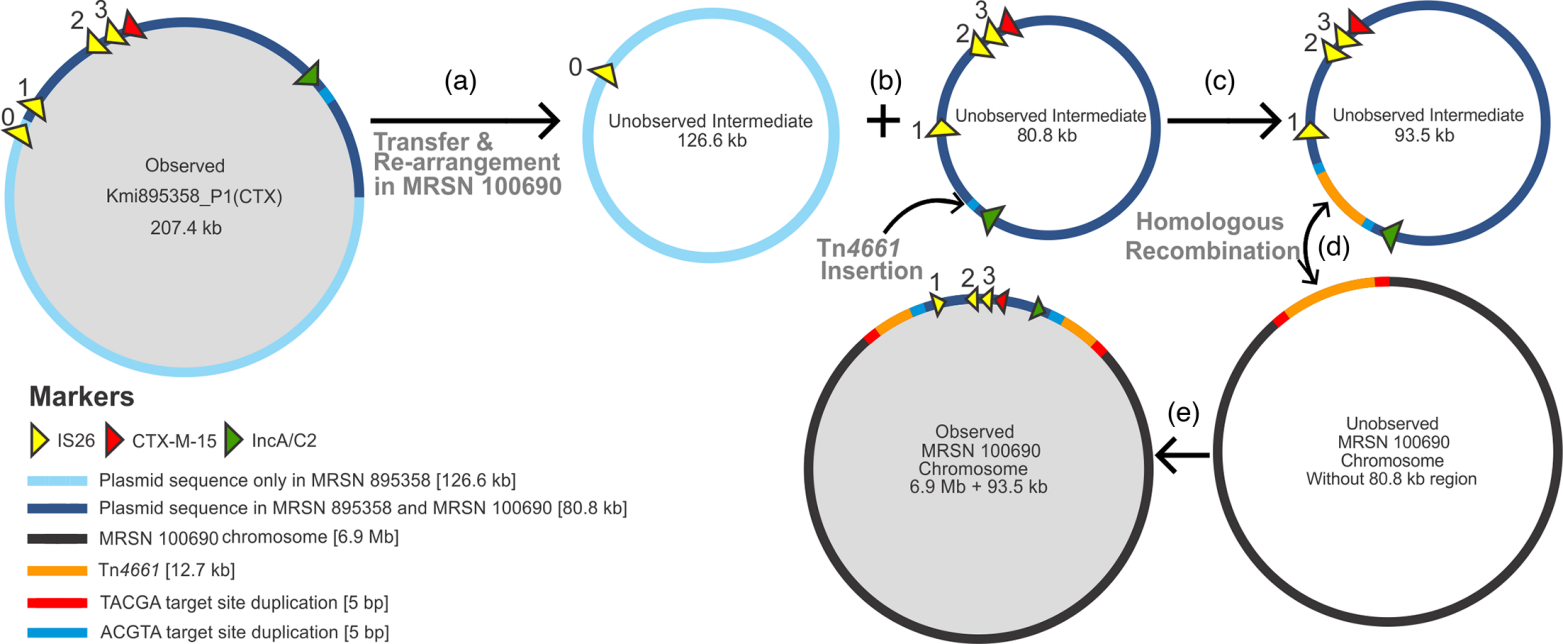
Proposed pathway of ESBL acquisition by 100 690. (**a**) Initial acquisition of Kmi895358_P1(CTX) by 100 690 (likely via conjugation) from 895 358. (**b**) Rearrangement of Kmi895358_P1(CTX) resulting in an 80.8 kb homologous region and a 126.6 kb non-homologous region. (**c**) A copy of Tn*4661* inserts into the 80.8 kb homologous region. (**d**) This Tn*4661* homologous region then integrates into the 100 690 chromosome via Tn*4661*-mediated recombination, (**e**) resulting in the observed structure containing *bla*_CTX-M-15_ within the 100 690 chromosome.

**Fig. 3.**
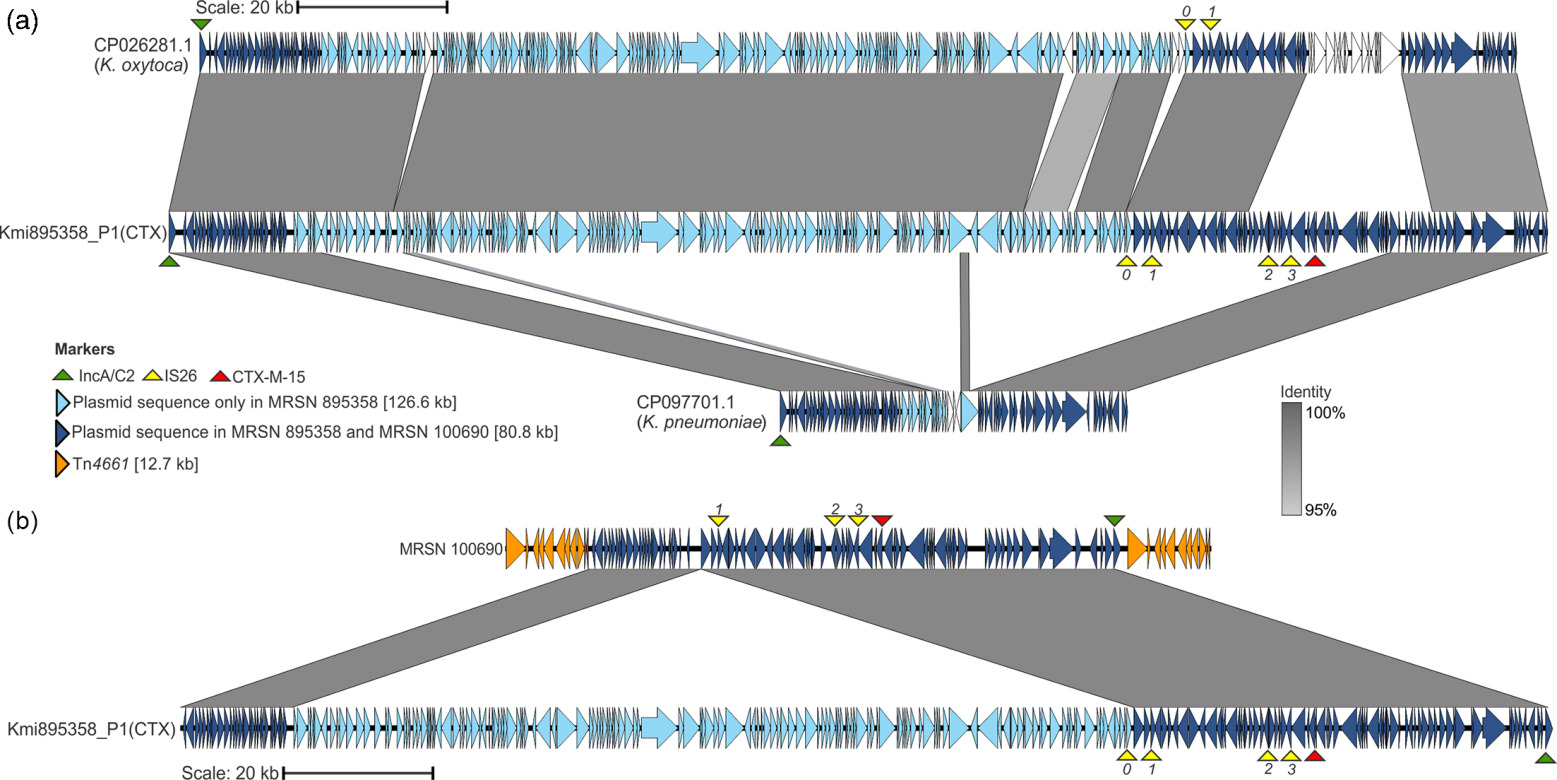
Mauve and pairwise alignment of (**a**) Kmi895358_P1(CTX) with CP026281.1 *K. oxytoca* and CP097701.1 *K. pneumoniae* segment; and (**b**) Kmi895358_P1(CTX) with the 100 690 chromosome. Plasmid sequence colour is identical to Fig. 2; 80.8 kb homologous region (dark blue), 126.6 kb non-homologous region (light blue), or Tn*4661* (orange). Percent shared identity between aligned sequences is depicted; aligned sequences with <95% shared identity are unshaded.

Long-read assembly produced two closed contigs (one chromosome, one plasmid) for *P. aeruginosa* 100 690 and four closed contigs (one chromosome, three plasmids) for *K. michiganensis* 895 358 (Tables1  2). In 100 690, *bla*_CTX-M-15_ was chromosomally bound (Figs 2e and 3b). In 895 358, it was located on a 207 430 bp plasmid designated Kmi895358_P1(CTX) (Table 2, Figs 2a and 3b). Mauve alignments of the 100 690 chromosome and Kmi895358_P1(CTX) showed a shared 80 796 bp region (Fig. 3b). The complete ~80.8 kb region in both isolates shared 100% nucleotide identity by pairwise alignment.

**Table 2.** Plasmid characteristics of strains in this study

Plasmid ID	Size (bp)	Replicon*	ARGs†
Kmi895358_P1(CTX)	207 430	IncA/C2	*aac(6')-Ib-cr5*, *ant(2'')-Ia*, *bla*_CTX-M-15_, *bla*_FOX-5_, *bla*_TEM-1_
Kmi895358_P2	195 656	IncFIB(K)	none
Kmi895358_P3	125 645	n/a	none
Psa100690_P1	58 873	n/a	none

1*Replicons type as determined by PlasmidFinder [17]. Inc, Incompatibility group. N/A: Not applicable.

2 †*In silico* derived plasmid-borne antimicrobial resistance genes (ARGs). A full table of all ARGs (chromosomal and plasmid) can be found in Table S2.

Notably, this region is absent from all other ST-621 *P. aeruginosa* from this facility. Apart from the shared 80.8 kb region, the remaining 126.6 kb region from the original Kmi895358_P1(CTX) did not align with any sequence in the 100 690 genome (Fig. 3b). On the 100 690 chromosome, the 80.8 kb shared region is flanked by a copy of Tn*4661* (Fig. 3b). The integrated second copy of Tn*4661* is a direct repeat of the original, which is ancestral to all ST-621. Both copies of Tn*4661* are oriented in the 5′ to 3′ direction and are the only two copies of Tn*4661* in the 100 690 genome (Figs 3b and 2e). Tn*4661* was not detected in the 895 358 genome (Fig. 3b).

All ST-621 isolates from MRSN repository (*n*=258) identified to be a part of the epidemic outbreak were found to have a copy of Tn*4661* with a target site duplication (TSD) sequence of TACGA approximately ~2.6 Mb downstream the origin of replication. 100 690 possessed two copies of Tn*4661* separated by the 80.8 Kb shared region containing *bla*_CTX-M-15_ (Fig. 2d). Of the 258 ST-621 isolates, it is the only one with two copies of Tn*4661*. The TSD sequence TACGA is observed outside both copies of Tn*4661* and a separate TSD sequence of ACGTA was observed on the inside of both Tn*4661* copies (Fig. 2e). This indicates that the pathway of chromosomal integration of the 80.8 kb shared region involved homologous recombination of Tn*4661*.

### Expression of *bla*_CTX-M-15_ is identical in both strains

Absolute transcript levels of *bla*_CTX-M-15_ were indistinguishable in *K. michiganensis* MRSN 895358 and *P. aeruginosa* MRSN 100690, irrespective of cefepime exposure (Fig. S2). *bla*_CTX-M-15_ absolute transcript levels were on average 31.5-fold higher (range: 9.5–92) than the reference genes *rpoD* and *dnaB*.

### Tn*4661* prevalence in MRSN and NCBI repositories

Tn*4661* is commonly found in *P. aeruginosa* genomes [21]. A blast analysis of 667 complete *P. aeruginosa* genomes from NCBI identified 98 with Tn*4661*. In contrast, a blast against 2291 complete *Klebsiella* sp. genomes found no copies of Tn*4661*. All 258 ST-621 *P. aeruginosa* draft genomes from Facility A [9] possessed Tn*4661*.

### Proposed molecular mechanism for *bla*_CTX-M-15_ acquisition and chromosomal integration in 100690

Based on the sequence data presented, the acquisition and chromosomal integration of the 80.8 kb region containing *bla*_CTX-M-15_ in *P. aeruginosa* 100 690 likely occurred through a mechanism like the following. Initially, Kmi895358_P1(CTX) from *K. michiganensis* 895 358 was acquired by 100 690, likely via conjugation (Fig. 2a). Once acquired, it underwent rearrangement to yield the 80.8 kb homologous sequence. Subsequently, Tn*4661* from the chromosome interacted with the ACGTA target site on the 80.8 kb shared region (Fig. 2b), leaving an ACGTA TSD sequence and a copy of Tn*4661* within the 80.8 kb shared region (Fig. 2c) to yield a 93.5 kb unobserved plasmid intermediate. Finally, homologous recombination of the Tn*4661* on the unobserved plasmid intermediate with the copy of Tn*4661* on the chromosome occurred (Fig. 2d), integrating the 80.8 kb fragment into the chromosome and generating the TSD in the process, along with the second copy of Tn*4661* (Fig. 2e).

### NCBI sequence similarities to the 207.4 kb donor plasmid

A blast query of 895358’s 126.6 kb non-homologous region to a custom database of 2291 *Klebsiella* sp. (2053 *K*. *pneumoniae*, 51 *K*. *michiganensis*, 57 *K*. *oxytoca*, 20 *K*. *grimontii*) genomes revealed that the plasmid backbone can be identified in 5 *K*. *pneumoniae*, 2 *K*. *oxytoca*, and 1 *K*. *grimontii* isolates (Table S3). In particular, the 126.6 kb non-homologous region shared high similarities (99% alignment fraction, 99.96% nucleotide identity) with CP026281.1, found in a *K. oxytoca* genome (Fig. 3a). Plasmid CP026281.1 possesses the same IncA/C2 replicon as Kmi895358_P1(CTX). A blast query of 895358’s 80.8 kb homologous region to *Klebsiella* sp. and *P. aeruginosa* genomes yielded no hits with significant coverage. The best hit was 51.8% coverage at a 99.98% identity to contig CP097701.1, belonging to a *K. pneumoniae* genome (Fig. 3a). This alignment had identical gene synteny with the 80.8 kb homologous region and both share the IncA/C2 replicon. However, CP097701.1 does not possess the three IS26 array, nor the *bla*_CTX-M-15_ ESBL (Fig. 3a).

## Discussion

Infections caused by CTX-M-producing pathogens are of clinical concern and incident cases are often escalated in infection control protocols. Active surveillance serves to quickly identify outbreaks in communities or clinical environments and can reduce dissemination of ESBL ARG determinants. The past two decades of surveillance have largely attributed CTX-M carriage to Enterobacterales, particularly *E. coli* and *Klebsiella* species [22]. However, recent reviews have cautioned the emergence of ESBLs in species from different taxonomic families, such as *P. aeruginosa* [23]. The precise mechanisms involved in ESBL acquisition by these emerging species are complicated and remain elusive.

As depicted in this study, inter-species horizontal plasmid transfer is one possible conduit, and this can occur both within a patient [24] and in the clinical environment [25]. We detected an identical 80.8 kb fragment observed in both a ST-621 *P. aeruginosa* and a ST-41 *K. michiganensis*. In *P. aeruginosa*, the fragment is chromosomally bound and in *K. michiganensis* is plasmid bound, constituting ~40% of the length of an observed 207.4 kb plasmid [Kmi895358_P1(CTX)] originally found in the *K. michiganensis* isolate. Though the exact details of the interaction between *P. aeruginosa* 100690 and *K. michiganensis* 895 358 can never be fully known, the available data suggests that plasmid transfer and subsequent transposition likely occurred within the hospital environment before transmission to the patients. First, the prolonged period of hospitalization for both patients before their respective positive cultures is a hallmark of nosocomial acquisition [26], indicating both patients likely acquired the bacteria from the environment. Second, environmental swabbing of the facility 3 months after P1 was discharged revealed that ST-621 *P. aeruginosa* was widespread across the facility, including in the sink drain of the room where both patients had stayed (Fig. 1). Notably, the strain (MRSN 103016) isolated during the environmental swabbing did not carry the 80.8 kb region, but other than that it was separated from 100 690 by just two SNPs across the entire genome. This strongly suggests they emerged from the same reservoir [9]. It is also important to note that while no *K. michiganensis* was recovered from the environmental swab, the recovery of *P. aeruginosa* was emphasized above other species from the heavily contaminated environmental swab and its presence may have been unnoticed [9]. Finally, despite multiple cultures from both patients during their prolonged hospital stays, no other related strains were cultured, supporting the hypothesis that the strains were acquired as cultured and that the genetic exchange occurred in the hospital environment.

Irrespective of whether the transfer occurred in the environment or the patient, the following pathway provides a convincing explanation for the acquisition of the *bla*_CTX-M-15_ in 100690: (i) the acquisition of Kmi895358_P1(CTX) by 100 690 from 895 358, (ii) intracellular plasmid rearrangement resulting in a 80.8 kb homologous region and a 126.6 kb non-homologous region, (iii) Tn*4661* insertion into the 80.8 kb homologous region, and (iv) integration of the homologous region by Tn*4661*-mediated recombination. Alternatively, the 80.8 kb homologous fragment could have been acquired by 100 690 with modifications to the Kmi895358_P1(CTX) occurring outside 100 690. This latter pathway involving genetic reduction before acquisition may be important to its persistence in the clinical setting through integration into different plasmid backbones typically attributed to *P. aeruginosa* and *Klebsiella* sp. In accordance, various ESBL and carbapenemase-producing organisms have been recovered from the hospital environment in surveillance studies, with the ARGs embedded within recognizable mobile genetic elements that are highly modular and found to be integrated into various plasmid backbones from different species [27,28].

Regardless of the origin and acquisition dynamics within ST-621 *P. aeruginosa*, the integration of ARGs in the *P. aeruginosa* genome is notably complex, with *bla*_CTX-M_ ESBL incidence rates remaining low and our understanding of their acquisition still incomplete. Despite this knowledge gap, the impact of *bla*_CTX-M-15_ in 100 690 is undeniably a clinical burden, rendering the strain non-susceptible to nearly all tested β-lactam antibiotics. Notably, absolute mRNA transcript levels of *bla*_CTX-M-15_ were indistinguishable in both strains, irrespective of the presence of cefepime in the growth media, indicating the gene is constitutively active in both species under the conditions tested in this experiment. As a result, concurrent clinical and environmental hospital surveillance is imperative to uncover species such as the ones depicted in this study, which possess the potential to precipitate future challenging outbreaks.

## supplementary material

10.1099/mgen.0.001303 Uncited Fig. S1.

10.1099/mgen.0.001303 Uncited Fig. S2.

10.1099/mgen.0.001303 Uncited Table S1.

## Abbreviations

ARG: antimicrobial resistance gene
CTX-M: cefotaxime-hydrolyzing β-lactamase isolated in Munich;
ESBL: extended-spectrum β-lactamase
HAI: healthcare-acquired infections
IS: insertion sequence
MDR: multidrug resistance
NCBI: National Center for Biotechnology Information
ST: sequence type
Tn: transposon
TSD: target site duplication
