# Supplementary material for: Tn4661-mediated transfer of blaCTX-M-15 from Klebsiella michiganensis to an outbreak clone of Pseudomonas aeruginosa
Source: Microb Genom. 2024 Oct 16;10(10):001303. doi: 10.1099/mgen.0.001303 (PMC11482538; doi:10.1099/mgen.0.001303)

Supplemental Figure S1. Schematic of plasmid Kmi895358_P1(CTX). Circular representation of plasmid Kmi895358_P1(CTX) from *K. michiganensis* MRSN 895358. The Origin of replication (ori) is indicated, and the position of antimicrobial resistance genes marked accordingly. The 80.8 kb region that is found in the chromosome of *P. aeruginosa* MRSN 100609 is depicted with green shading.

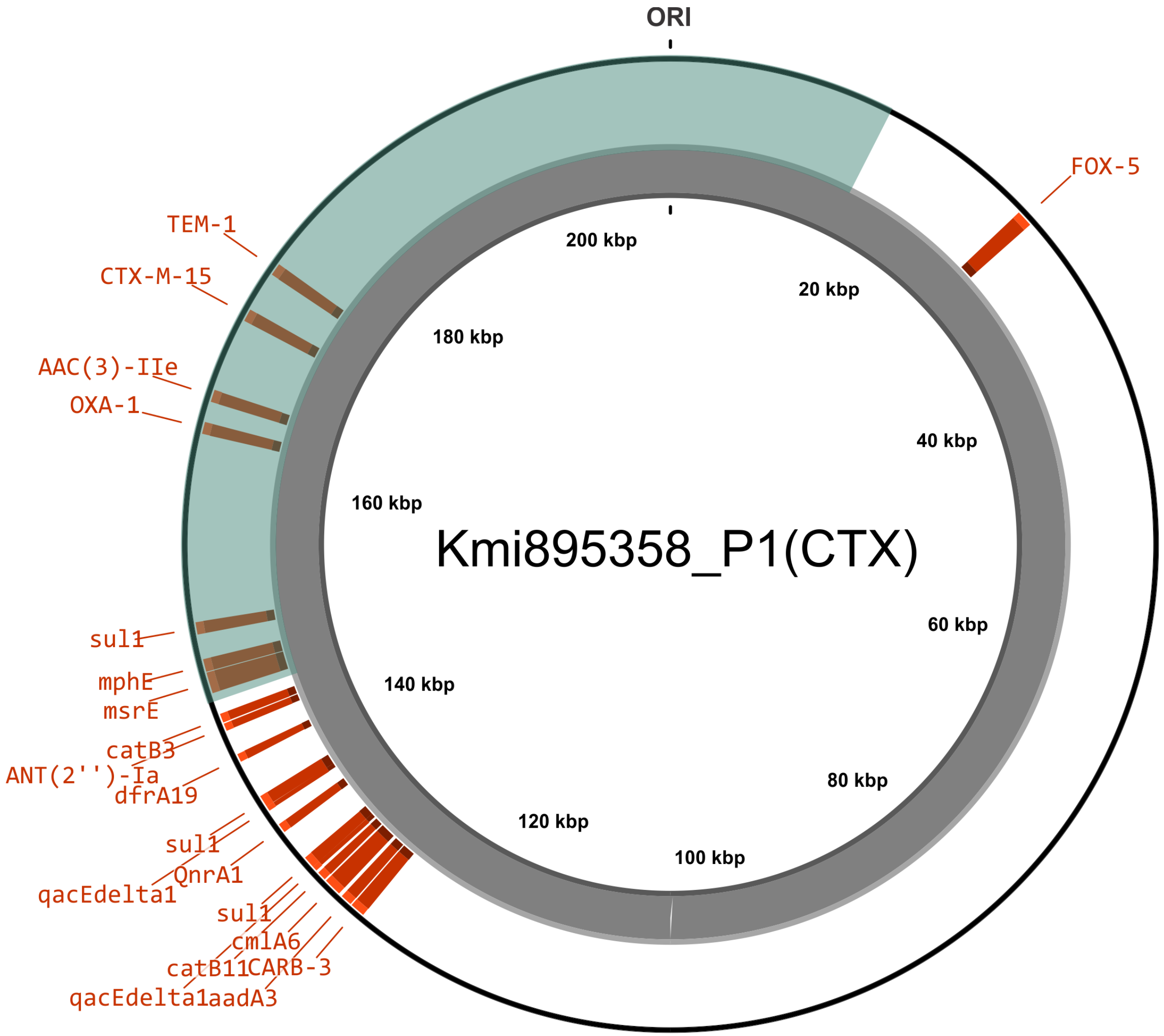

Supplement: Uncited Fig. S1. [file mgen-10-01303-s001.pdf]
