# Supplementary material for: Tn4661-mediated transfer of blaCTX-M-15 from Klebsiella michiganensis to an outbreak clone of Pseudomonas aeruginosa
Source: Microb Genom. 2024 Oct 16;10(10):001303. doi: 10.1099/mgen.0.001303 (PMC11482538; doi:10.1099/mgen.0.001303)

Supplemental Figure 2. Transcription analysis of *bla*$_{CTX-M-15}$ in *K. michiganensis* MRSN 895358 and *P. aeruginosa* MRSN 100690 in the presence and absence of cefepime. Normalized, log-transformed messenger RNA (mRNA) levels are indicated. Data represent the average from 3 independent experiments and error bars represent the standard deviation. FEP, cefepime; PBS, phosphate buffered saline.

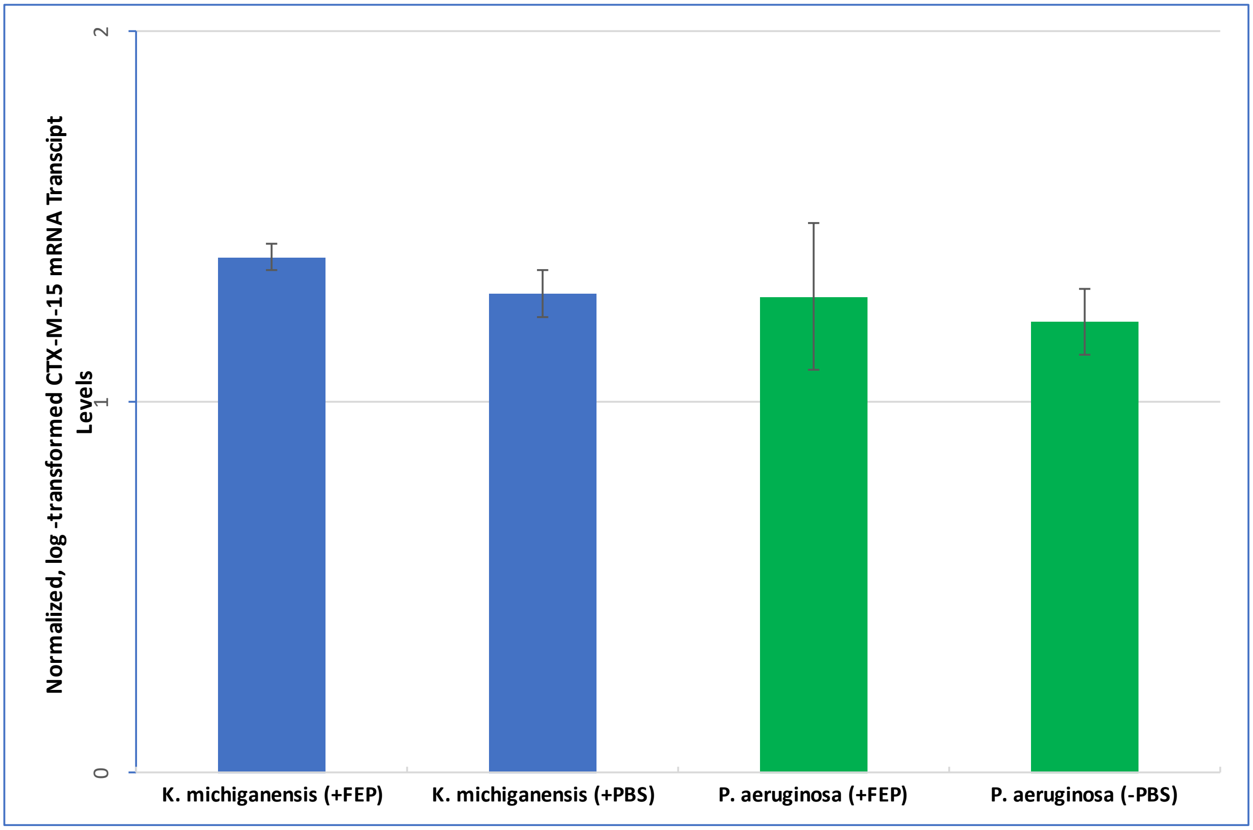

Supplement: Uncited Fig. S2. [file mgen-10-01303-s002.pdf]
